# Nonsteroidal anti-inflammatory drugs and acetaminophen ameliorate muscular mechanical hyperalgesia developed after lengthening contractions via cyclooxygenase-2 independent mechanisms in rats

**DOI:** 10.1371/journal.pone.0224809

**Published:** 2019-11-06

**Authors:** Tetsuhiro Shimodaira, Shigeo Mikoshiba, Toru Taguchi

**Affiliations:** 1 Pharmaceutical Research Laboratories, Lion Corporation, Odawara, Japan; 2 Department of Physical Therapy, Niigata University of Health and Welfare, Kita-ku, Niigata, Japan; 3 Institute for Human Movement and Medical Sciences, Niigata University of Health and Welfare, Kita-ku, Niigata, Japan; Temple University, UNITED STATES

## Abstract

Nonsteroidal anti-inflammatory drugs and acetaminophen are cyclooxygenase inhibitors commonly used as symptomatic medicines for myofascial pain syndrome. Using the selective inhibitors celecoxib and zaltoprofen, cyclooxygenase-2 has been shown to be involved in the initiation, but not the maintenance, of muscular mechanical hyperalgesia induced by lengthening contractions, which serves as a useful model for the study of myofascial pain syndrome. The effect of other cyclooxygenase-2 inhibitors, such as acetylsalicylic acid, ibuprofen, loxoprofen sodium, and acetaminophen, on muscular mechanical hyperalgesia during maintenance has not been studied. Here, we examined the analgesic effects of the nonsteroidal anti-inflammatory drugs and acetaminophen on the model. Consistent with previous studies, mechanical withdrawal threshold of the muscle was significantly decreased and reached its lowest level 24 h after lengthening contractions. Celecoxib had no effect on muscular mechanical hyperalgesia, when orally administered 24 h after lengthening contractions. In contrast, acetylsalicylic acid, ibuprofen, loxoprofen sodium, and acetaminophen increased the withdrawal threshold, which had decreased by lengthening contractions, in a dose-dependent manner. These results demonstrate the analgesic actions of nonsteroidal anti-inflammatory drugs and acetaminophen in the maintenance process of lengthening contraction-induced muscular mechanical hyperalgesia, which may occur through cyclooxygenase-2 independent mechanisms.

## Introduction

Myofascial pain syndrome (MPS) is a debilitating condition characterized by pain in the muscle and fascia. MPS may be associated with a variety of clinical conditions, such as lower back pain, stiff neck, tension-type headache, and temporomandibular joint dysfunction [1]. It is characterized by the presence of myofascial trigger points (MTrPs), which are localized spots in the taut band of the muscle with decreased nociceptive threshold, mechanical hyperalgesia, and referred pain induced by mechanical irritation of the MTrPs [2]. Although there are several hypotheses for the induction mechanisms, MTrPs are assumed to be created in specific muscles subjected to overload or sustained contractions [3–5]. To understand the mechanism and develop an effective treatment, a putative animal model of MPS is needed.

Previous studies have suggested that an animal model of MPS can be established by lengthening contraction (LC) of the muscle [6,7]. LC is a type of muscle contraction where the contracting muscle is simultaneously stretched. Although this LC model was originally reported as a useful tool for the study of delayed onset muscle soreness (DOMS) [8], it has some similarity with the clinical features of MPS. First, the LC model is characterized by mechanical hyperalgesia in the over-exercised muscle, which is the most striking characteristic of MPS. Second, the model exhibits taut band-like muscle hardening which is palpable in the exercised muscle after repetitive LC in rabbits and humans [6,9]. Third, MTrP-like localized sensitive spots were observed after LC, especially in depth of the fascia in the palpable taut bands. [6,9]. Fourth, severe myofiber damage and the subsequent inflammation were not discernible in the entire muscle that underwent LC at least by light microscopic analysis [10]. This histological observation aligns with findings that the upregulation of inflammatory mediators does not occur in the latent MTrPs [11].

Two neurotrophic factors—nerve growth factor (NGF) and glial cell line-derived neurotrophic factor (GDNF)—play pivotal roles in mechanical hyperalgesia in the rat LC model [10,12–14]. Despite a lack of scientific evidence supporting neurotrophic factor upregulation in clinical MTrPs, repeated intramuscular injections of NGF in humans induced localized mechanical hyperalgesia and enlargement of the referred pain area evoked by pressure stimulus, similar to those seen in clinical MTrPs [15]. Thus, an LC model with an upregulated neurotrophic factor can be used to study MPS.

In the rat LC model, orally administering the cyclooxygenase-2 (COX-2) selective inhibitors, celecoxib and zaltoprofen, one hour before exercise completely suppressed the development of muscular mechanical hyperalgesia. However, when administered 2 days after LC, amelioration did not occur [14]. This finding demonstrated the involvement of COX-2 only in the initiation and not the maintenance of muscular mechanical hyperalgesia. The analgesic effects of other COX-2 inhibitors, such as acetylsalicylic acid (ASA), ibuprofen, loxoprofen sodium (LOX), and acetaminophen, have not been investigated.

Nonsteroidal anti-inflammatory drugs (NSAIDs) and acetaminophen are major drugs for acute pain, and commonly used asymptomatic medication for MPS [16,17]. Some studies have provided strong evidence to support the use of oral NSAIDs for acute pain treatment such as acute lower back pain and postoperative pain [18,19]. However, the effects of orally administering NSAIDs and acetaminophen on MPS have not been examined [17], including their analgesic mechanisms.

Although NSAIDs typically act via COX-2 inhibition [20–22], other pharmacological targets, such as inducible nitric oxide synthase, peroxisome proliferator-activated receptor gamma, inhibitory kappa B kinase-beta, acid-sensing ion channels (ASICs), transient receptor potential vanilloid 1 (TRPV1), and caspases, have been suggested to counteract pain and inflammation [23–28]. Acetaminophen does not have significant anti-inflammatory and COX-2 inhibitory effects in peripheral tissues. Instead, it is assumed to cause analgesia and anti-pyresis via COX-2 inhibition in the central nervous system [29–32], such as the serotonergic descending pathway and the opioid system [33–37]. Recently, the active metabolite of acetaminophen, N-acylphenolamine (AM404), was shown to be involved in the analgesic effect of acetaminophen through TRPV1 in the spinal cord [38].

Using a rat model of LC-induced MPS, the current study was performed to examine the analgesic effect of NSAIDs and acetaminophen on muscular mechanical hyperalgesia, and the involvement of COX-2 in their analgesic mechanisms. Our findings suggest that COX-2 independent mechanisms cause the analgesic actions of NSAIDs and acetaminophen in the maintenance phase of LC-induced MPS.

## Results

### Experiment I: Time course of LC-induced muscular mechanical hyperalgesia

**Fig 1:**
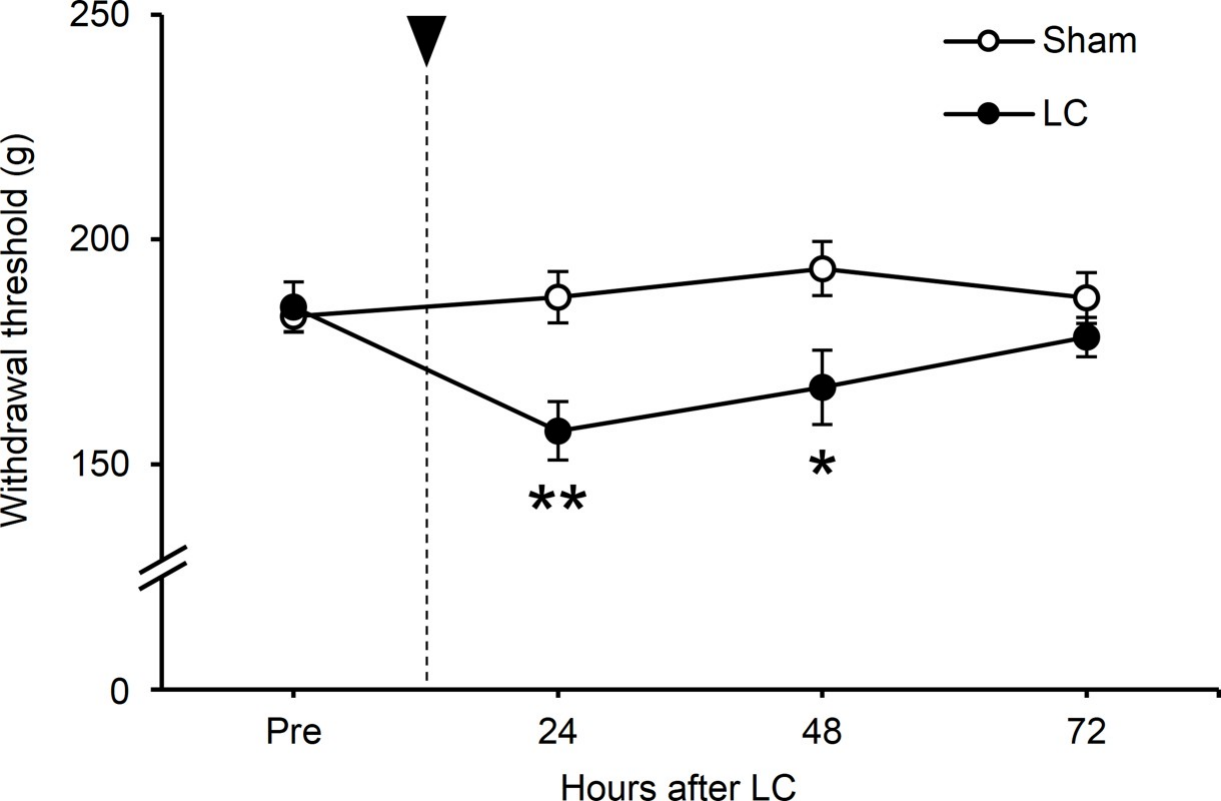
Induction of muscular mechanical hyperalgesia using lengthening contractions (LC). Changes in the mechanical withdrawal threshold of the exercised muscle. Black arrow indicates the time point of LC. Abscissa: hours after LC. Ordinate: muscular mechanical withdrawal threshold (in grams). A significant reduction in the threshold occurred at 24 and 48 h after LC (n = 6 rats in each group, *P < 0.05 and **P < 0.01 vs. Sham, two-way repeated measures ANOVA followed by Student’s t-test).

To determine the experimental time point for drug efficacy, mechanical withdrawal threshold was measured daily until 3 days after LC or Sham (Fig 1). Withdrawal thresholds were found to decrease after LC. Two-way repeated measures ANOVA (analyzed parameters: time and treatment) revealed significant treatment effect (*F*_1,10_ = 6.207; *P* < 0.05) and non-significant time effect (*F*_3,30_ = 2.885; *P* = 0.052), with a significant interaction between the two factors (*F*_3,30_ = 5.905; *P* < 0.01). Post-hoc analysis revealed that the withdrawal threshold in the LC group significantly decreased 24 and 48 h after LC, as compared to Sham (*P* < 0.05, Student’s t-test), and was at its lowest 24 h after LC. Hence, we chose to examine the analgesic effect of the drugs 24 h after LC through subsequent experiments.

### Experiment II: Involvement of COX-2 in the generation and maintenance of the LC-induced muscular mechanical hyperalgesia

Celecoxib, a COX-2 selective inhibitor, suppresses development of muscular mechanical hyperalgesia, when orally administered 1 h before LC, but not 48 h after LC [14]. Hence, we investigated the involvement of COX-2 in the earlier phase (i.e., 24 h after LC), and attempted to replicate the preventive effect of COX-2 inhibition on the development of LC-induced mechanical hyperalgesia.

**Fig 2:**
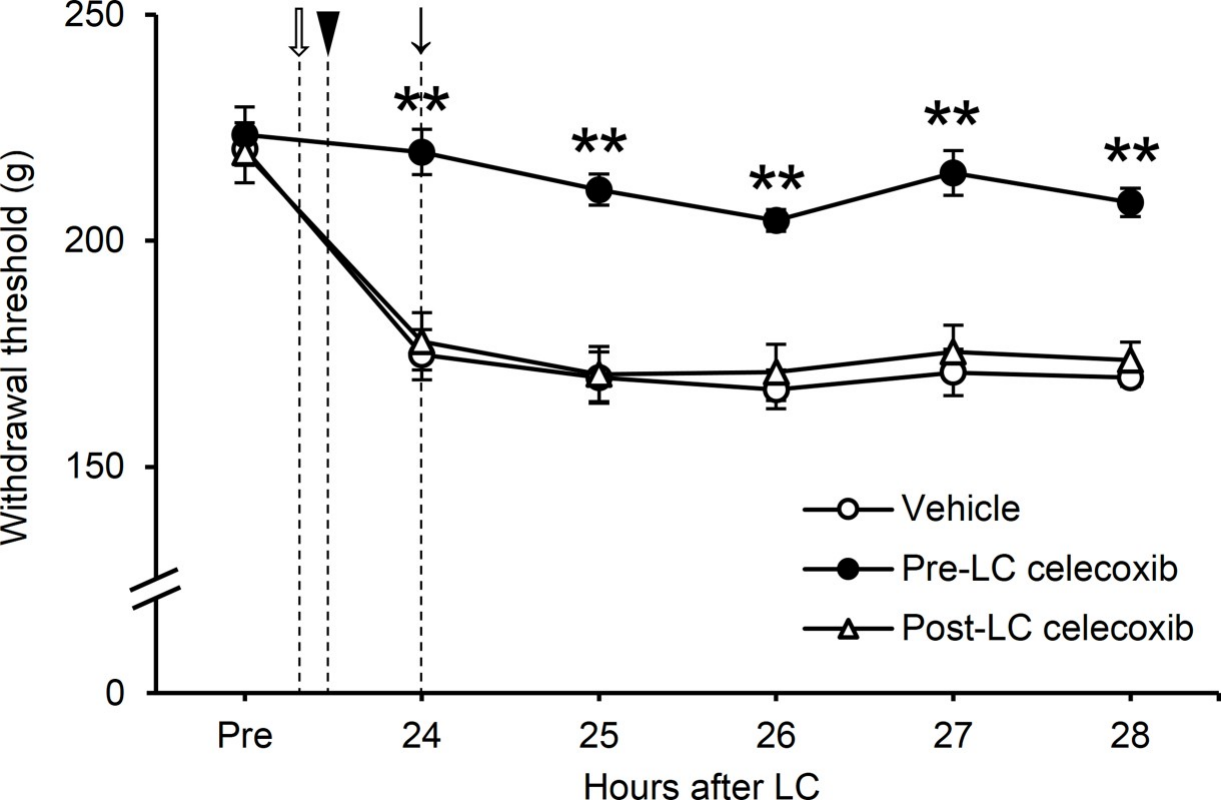
Effects of celecoxib on the development and maintenance processes of lengthening contractions (LC)-induced muscular mechanical hyperalgesia. Celecoxib (10 mg/kg, p.o.) was administered either 1 h before (Pre-LC celecoxib, white arrow) or 24 h after LC (Post-LC celecoxib, black arrow). Baseline withdrawal thresholds (Pre) were measured 1 day before LC. An arrowhead indicates the time point of LC. The development of mechanical hyperalgesia was blocked when celecoxib was administered 1 h before LC in the Pre-LC celecoxib group, but not when administered 24 h after LC in the Post-LC celecoxib group (n = 6 rats in each group, **P < 0.01 vs. vehicle and Post-LC celecoxib, two-way repeated measures ANOVA followed by Tukey’s post-hoc test).

Administering celecoxib 1 h before LC (Pre-LC celecoxib) prevented mechanical hyperalgesia development, agreeing with the results of a previous study [14]. However, when administered 24 h after LC (Post-LC celecoxib), celecoxib did not change the decrease in the mechanical withdrawal threshold. Two-way repeated measures ANOVA (analyzed parameters: time and treatment) revealed significant treatment (*F*_2,15_ = 24.21; *P* < 0.01) and time effects (*F*_5,75_ = 59.96; *P* < 0.01), with a significant interaction between the two factors (*F*_10,75_ = 10.25; *P* < 0.01). Post-hoc analysis showed that the mechanical withdrawal threshold in the Pre-LC celecoxib group remained significantly higher than the post-LC celecoxib and the vehicle group (*P* < 0.01, Tukey’s test, Fig 2).

### Experiment III: Dose-dependent effect of NSAIDs and acetaminophen on muscular mechanical hyperalgesia

**Fig 3:**
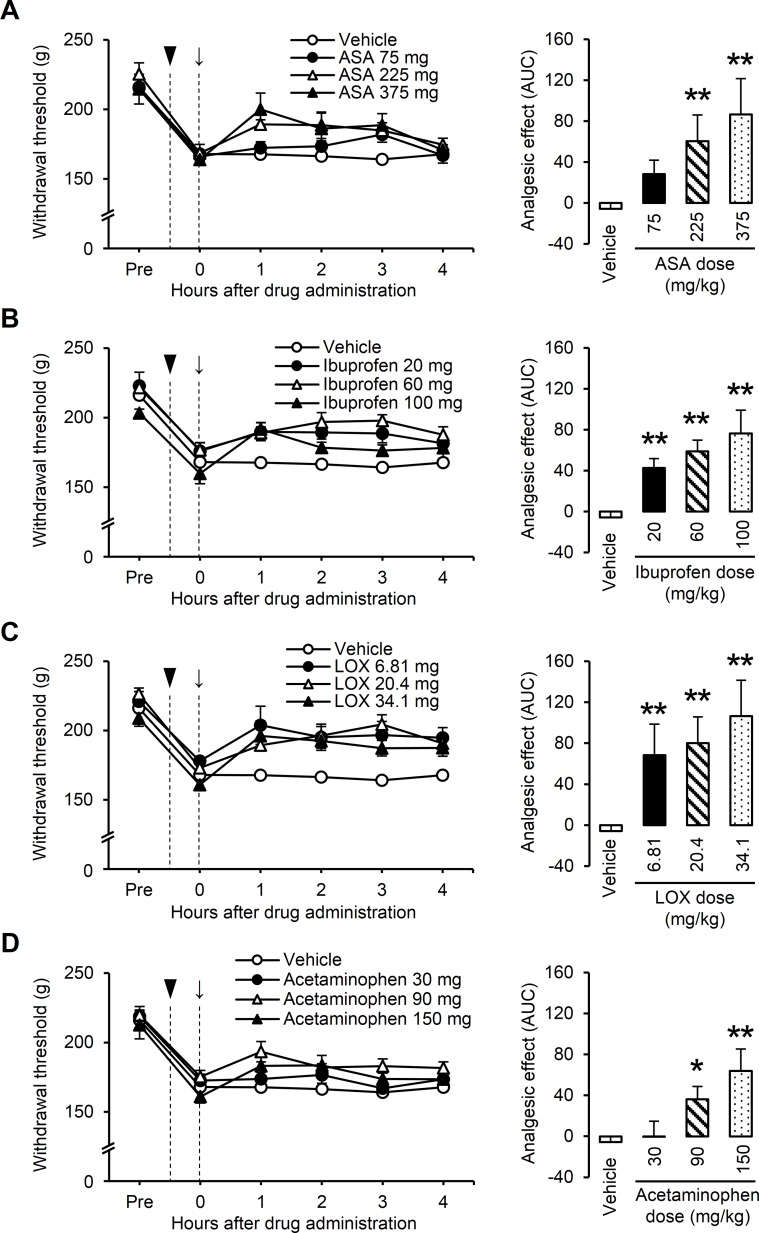
Dose-dependent analgesic effects of NSAIDs and acetaminophen on muscular mechanical hyperalgesia when developed by lengthening contractions (LC). Effect of (A) acetylsalicylic acid (ASA 75, 225, or 375 mg/kg), (B) ibuprofen (20, 60, or 100 mg/kg), (C) loxoprofen sodium (LOX 6.8, 20.4, or 34.1 mg/kg) and (D) acetaminophen (30, 90, or 150 mg/kg). All drugs were administered orally. Arrow and arrowhead indicate time point of drug administration and LC, respectively. Baseline withdrawal thresholds (Pre) were measured 1 day before LC and drugs were administered 24 h after LC. Graphs on the right side show the magnitude of analgesia with an index of the area under the time course curves (AUC) of the withdrawal thresholds. ASA, ibuprofen, LOX, and acetaminophen dose-dependently increased AUC (n = 6 rats for each concentration of the drugs, and n = 36 rats pooled in the vehicle group so that the larger sample size was obtained, *P < 0.05 and **P < 0.01, vs. vehicle, one-way ANOVA followed by Dunnett’s post-hoc test). Data of the vehicle group is identical for Fig 3A–3D.

We examined the analgesic effect of ASA, ibuprofen, LOX, and acetaminophen on muscular mechanical hyperalgesia when developed 24 h after LC. All tested drugs increased the mechanical withdrawal threshold, which had decreased after LC, in a dose-dependent manner (Fig 3A–3D). ASA, ibuprofen, LOX, and acetaminophen increased analgesic effect (AUC) with a significant treatment effect found by one-way ANOVA (ASA: *F*_3, 50_ = 10.029, *P* < 0.01; ibuprofen: *F*_3, 50_ = 16.364, *P* < 0.01; LOX: *F*_3, 50_ = 21.470, *P* < 0.01; and acetaminophen: *F*_3, 50_ = 8.139, *P* < 0.01). Post-hoc analysis revealed a significant increase in mechanical withdrawal threshold for the following doses of tested drugs: 225 and 375 mg/kg for ASA (*P* < 0.01, Dunnett’s test, Fig 3A); 20, 60, and 100 mg/kg for ibuprofen (*P <* 0.01, Dunnett’s test, Fig 3B); 6.81, 20.4 and 34.1 mg/kg for LOX (*P <* 0.01, Dunnett’s test, Fig 3C); and 90 and 150 mg/kg for acetaminophen (*P* < 0.05 and *P <* 0.01, respectively, Dunnett’s test, Fig 3D).

## Discussion

Using a rat model of LC-induced MPS, in the present study, we demonstrated that NSAIDs and acetaminophen, but not celecoxib, ameliorated muscular mechanical hyperalgesia. These results indicate that COX-2 is not involved in the maintenance of mechanical hyperalgesia when developed by LC, and NSAIDs and acetaminophen may act through mechanisms other than COX-2 inhibition.

As reported previously, muscle damage and the resulting inflammation, in which COX-2 is involved, are not required for mechanical hyperalgesia when developed after LC [10]. Instead of COX-2, neurotrophic factors (NGF and GDNF), TRP channels (TRPV1 and TRPV4) and ASICs play critical roles in maintaining muscular mechanical hyperalgesia in the LC model [12] (for review).

In the present study, a single oral administration of NSAIDs and acetaminophen increased the mechanical withdrawal threshold of the muscle within 1 h, and the analgesic effect continued for at least 4 h during the observation period. Plasma concentrations of NSAIDs and acetaminophen have been reported to reach their peaks within 1 h when administered orally to rats, and their half-life decay range from 30 min to 3 h [39–42]. The time course of analgesia was quite similar to the plasma concentration of NSAIDs and acetaminophen. Thus, the drugs tested in the present study might have inhibited the activities of ion channels, such as TRPV1, TRPV4, and ASICs that are expressed in primary sensory neurons, thereby causing analgesia as observed in this study.

The COX-independent pharmacological action of COX inhibitors has been reported, and the COX inhibitors tested in the present study were demonstrated to modulate TRP and ASIC channel activities [26,27]. TRPV1 is sensitive to capsaicin, noxious heat, and protons [43] (for review), and is involved in muscular mechanical hyperalgesia induced by LC [44,45]. ASA reduces capsaicin-induced pain [46] and heat-evoked inward current of primary nociceptive neurons [47]. Maurer *et al*. reported that ASA increased tachyphylaxis of TRPV1 to capsaicin stimuli when applied repetitively [27]. This effect was independent of COX inhibition since inhibition occurs within 30 s of pre-incubation, whereas reduction of prostaglandin synthesis takes several minutes [48]. The inhibitory concentration of TRPV1 by ASA (1 μM) is far less than the half maximal inhibitory concentration (IC_50_) of ASA on COX-2 (IC_50_ = 35.5 μM). In rats, peak plasma concentration (C_max_) of ASA in response to a single oral dose of ASA (40 mg/kg) is ~54 μM [40]. Thus, TRPV1 inhibition can occur with the dose used in the present study.

Ibuprofen was reported to have a weak but significant analgesic effect on a rat model of capsaicin-induced mechanical hyperalgesia at a single oral dose of 61.8 mg/kg, but not at 3–30 mg/kg celecoxib [49]. Protein kinase C (PKC) has been reported to be involved in TRPV1 sensitization by NGF [50]. Acetaminophen, but not celecoxib and ibuprofen, inhibits PKC epsilon (PKCε) translocation to the cell membrane of DRG neurons at a concentration of 10 μM [51]. The C_max_ of acetaminophen in rats is ~104 μM when an oral dose of 20 mg/kg is administered [42]. Thus, modulation of PKCε activity with acetaminophen could occur in this study. The inhibitory effect of LOX on TRPV1, and the inhibitory effect of these COX inhibitors on TRPV4 has been largely unexplored. However, PKCε has been implicated in modulating TRPV4 activity [52–54]. Based on these findings, modulation of TRP channel activity likely explains the analgesic effect of ASA, ibuprofen, and acetaminophen observed in this study.

ASICs have at least six subunits (1a, 1b, 2a, 2b, 3, and 4). ASIC3 and ASIC1 have been reported to play a critical role in the development of acidic saline-induced muscular hyperalgesia [55–57]. ASIC3 has been shown to be involved in the LC-induced muscular mechanical hyperalgesia [58]. Voilley *et al*. reported that ASA and salicylic acid (one of the main metabolites of ASA) inhibit proton-evoked inward current in COS cells expressing ASIC3 in whole-cell patch-clamp experiments [26]. However, other NSAIDs, such as piroxicam, etodolac, nimesulide, naproxen, indomethacin, and acetaminophen, had no effect. When ASA is administered orally to rats at a dose of 20 mg/kg, the IC_50_ of salicylic acid on ASIC3 is 260 μM while its C_max_ is ~143 μM [59]. Thus, ASA may inhibit ASIC3 activity using the dose tested in the present study. Ibuprofen has been demonstrated to inhibit ASIC1a activity at 500 μM [26] via allosteric binding to a crucial site in the agonist transduction pathway [60]. In rats, C_max_ of ibuprofen is ~378 μM when a single oral dose of 20 mg/kg is administered [39]. Thus, ibuprofen might inhibit muscular mechanical hyperalgesia via ASIC1a.

Acetaminophen has been shown to possess analgesic mechanisms via the central nervous system [30,38,61]. Acetaminophen activates the descending serotonergic and cannabinoid system. However, the drug doses examined in previous studies (300–1000 mg/kg [35,62]) were at least two-fold greater than those used in this study. The acetaminophen metabolite, AM404, has been reported to induce analgesia by activating TRPV1 in the spinal dorsal horn with a clinically-relevant cerebrospinal fluid concentration of 30 μM [38]. In the present study, acetaminophen may therefore act via the descending pain inhibitory pathways and TRPV1 activation in the spinal dorsal horn.

The LC model used for the study of MPS had some limitations. First, the duration of mechanical hyperalgesia in the muscle was rather short (i.e., for 3 days after LC) when compared to that observed in the clinic with MPS patients. Second, the biochemical features of active MTrPs, as reported previously [11], were not characterized in the LC model. By using a microdialysis technique in patients, the concentration of pain-related biochemicals, such as protons, substance P, calcitonin gene related peptide, tumor necrosis factor-alpha, interleukin (IL)-1β, IL-6, IL-8, serotonin, and noradrenaline, was reported to increase in an area of active MTrPs with spontaneous pain, but not in latent MTrPs without spontaneous pain [11]. Third, the existence of referred pain, which is one of the most important symptoms of MPS, was not confirmed in the rat LC model.

In summary, we demonstrated for the first time that the oral administration of NSAIDs and acetaminophen ameliorated muscular mechanical hyperalgesia when developed after LC, and may have occurred through mechanisms other than COX-2 inhibition. Further studies are however required to understand the analgesic mechanism of the NSAIDs and acetaminophen.

## Methods

### Animals

Male Sprague-Dawley rats (weight, 250–270 g; age, 8 weeks, SLC Inc, Sizuoka, Japan) were used in this study. Two rats were housed per cage in a clean room under a controlled temperature of 21–23°C and 12 h light/dark cycle (lights on at 07.00 h). Rats had access to food and water ad libitum but were fasted for 12 h prior to oral drug administration. The present study was approved by the Animal Care and Use Committee of LION Corporation and is in accordance with Guidelines for Proper Conduct of Animal Experiment from the Science Council of Japan. After the end of the experiment, rats were euthanized by CO_2_ gas inhalation.

### Lengthening contractions

**Fig 4:**
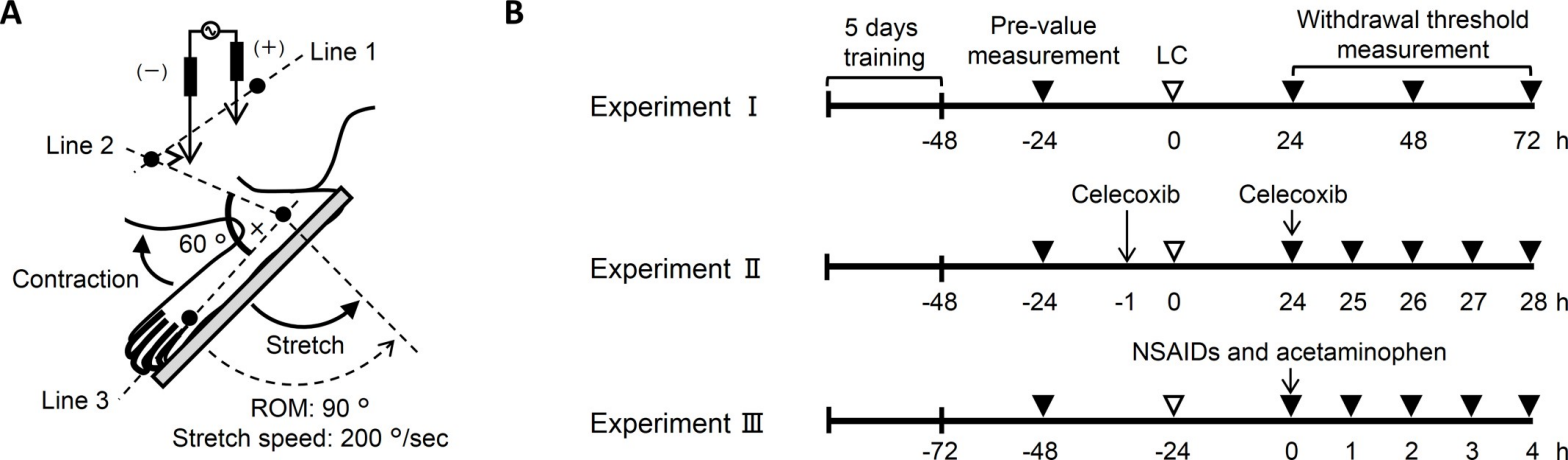
Lengthening contractions and experimental schedule. (A) Schematic illustration of the method for lengthening contractions (LC, modified from [10] with permission). LC was induced by electrical stimulation through a pair of needle electrodes, which were insulated except for the tips. A positive and negative electrode was inserted near the sciatic and peroneal nerve, respectively. Electronic currents were supplied via the needle electrodes to induce contraction of the ankle extensor muscles. In synchronization with contraction, the extensor muscles were stretched by rotating a foot plate, which was attached and fixed to the rat footpad. Stretch range of motion (ROM) was precisely defined based on three axes (see Methods for details). LC was performed with a ROM of 90° and angular velocity of 200°/s for a total of 50 repetitions. (B) Time schedule for Experiment I–III. Following training sessions for 5 days, pre-value was measured 24 h prior to LC. Measurements were repeatedly performed to determine the time course of muscular mechanical hyperalgesia (Experiment I), to examine analgesic effect of celecoxib (Experiment II), and to examine the efficacy of NSAIDs and acetaminophen (Experiment III). Open arrowhead: time point of LC, closed arrowheads: time points of threshold measurement, and arrows: time points of oral administration of drugs.

LC were applied with a protocol reported previously [10]. In brief, rats were anesthetized by inhalation of 1.5% isoflurane. LC was performed with a customized device (NDH-1; Bio Research Center, Nagoya, Japan) that enabled the control of LC stretch variables [63]. The left hindleg dorsiflexor muscles were repetitively contracted by electrically stimulating the common peroneal nerve with a pair of insulated needle electrodes, except for at the tips [8] (Fig 4A). Currents were supplied with an isolator (SS-203J; Nihon kohden Corp., Tokyo, Japan) connected to an electrical stimulator (SEN-3401; Nihon kohden Corp.). The parameters of electrical stimulation: current strength 3-fold greater than the twitch threshold (< 100 μA), frequency of 50 Hz, and pulse duration of 1 ms.

To stretch the contracting muscles during LC, the footpad of rats was fixed on a foot plate and its movement was synchronized with an electrical stimulator (Fig 4A). The range of motion (ROM) was precisely defined based on three axes: the femoral axis (between the third trochanter and lateral condyle of the femur), the fibular axis (between the head and lateral malleolus of the fibula), and the foot axis (along the base of the metatarsal). Joint angles of the knee and ankle were set at 90° and 60°, respectively. The central axis of ankle joint rotation was 3 mm in front of the lateral malleolus of the fibula. LC was performed 50 times with a ROM of 90° and angular velocity of 200°/s. Rats with muscle stretch alone (i.e., without contraction by electrical stimulation) served as the control (Sham).

### Behavioral pain test

Mechanical withdrawal threshold of the exercised muscle to pressure stimulus was measured with a method previously reported [10]. In brief, an electronic von Frey apparatus (No. 2391; IITC Inc, Los Angeles, CA, USA) was used to measure the threshold. Rats were restrained with a towel and a jacket was wrapped around their trunks to calm them; their legs were however allowed to move freely. Rats were treated gently during the experiments. A pusher with a flat tip (tip diameter: 3 mm) was applied to the belly of the exercised muscle on an anterior part of the leg through shaved skin. The intensity of the pressure that caused an escape reaction was defined as the withdrawal threshold. Training sessions were conducted for at least five days to increase the sensitivity of the test. Measurements were performed before, 24, 48, and 72 h after LC or Sham to determine the time course of muscular mechanical hyperalgesia (Experiment I, Fig 4B). To examine the efficacy of the analgesics, measurements were performed before, 24, 25, 26, 27, and 28 h after LC (i.e., before LC, and 0, 1, 2, 3, and 4 h after drug administration, Experiment II and III, Fig 4B). Measurements were repeated five times at more than 1-minute intervals, and mean value was taken as the threshold. Thus it usually takes for less than 10 minutes in each measurement. Six rats were assigned to each group. The experimenter was blinded to the drug and the concentration received by the rats.

### Drug administration

Celecoxib (10 mg/kg, Tokyo Chemical Industry Co., Tokyo, Japan) was used as a COX-2 selective inhibitor. Oral administration of this dosage significantly suppressed hyperalgesia and prostaglandin E2 production in a rat model of carrageenan-induced inflammatory pain [64]. Moreover, it was used in a previous study [14]. ASA (75–375 mg/kg, Yoshida Pharmaceutical Co., Saitama, Japan), ibuprofen (20–100 mg/kg, Shiratori Pharmaceutical Co., Chiba, Japan), LOX (6.8–34.1 mg/kg, Daiwa Pharmaceutical Co., Toyama, Japan), and acetaminophen (30–150 mg/kg, Iwaki Seiyaku Co., Tokyo, Japan) were the drugs used; their doses were determined using the maximum single oral dosages for non-prescription medicine in adult human subjects as approved by the Ministry of Health, Labor and Welfare in Japan. Oral analgesic dosages of ASA, ibuprofen, LOX, and acetaminophen in rat pain models varies from 100–400 mg/kg, 6.2–150 mg/kg, 1–30 mg/kg, and 200–1000 mg/kg depending on the model used, respectively [35,49,62,64,65]. Thus, the doses of all drugs used in the current study were within the range of previous works. All drugs tested were suspended in 5% gum Arabic and administered orally at 10 ml/kg. Solution of 5% gum Arabic without drugs served as a control in the vehicle group. Celecoxib was administered either 1 h before LC or just after the behavioral tests 24 h after LC. ASA, ibuprofen, LOX, and acetaminophen were administered orally just after mechanical threshold measurement 24 h after LC. As the time course for the changes in threshold differed among drugs and doses, the analgesic effect of the NSAIDs and acetaminophen was quantified as the area under the curve (AUC) of the withdrawal threshold calculated from the change in threshold (g) by subtracting the threshold at four time points (i.e., 1, 2, 3, and 4 h after drug administration) from the threshold 24 h after LC in each rat.

### Statistical analyses

Data are presented as mean ± SEM. Changes in withdrawal threshold after LC were analyzed by two-way repeated measures analysis of variance (ANOVA). When the sphericity assumption was not met, Greenhouse-Geisser correction was used to modify the degree of freedom. Post-hoc analysis was performed when appropriate. Student’s post-hoc t-test was used to compare the Sham and LC group at single time points. Tukey’s multiple comparison post-hoc test was used to compare celecoxib to the vehicle. Changes in AUC after NSAIDs and acetaminophen administration were compared to vehicle using one-way ANOVA followed by Dunnett’s multiple comparison post-hoc test. Differences were considered significant when *P* was < 0.05.

## Supporting information

S1 Table Withdrawal threshold in lengthening contraction and sham group. (XLSX)

S2 Table Withdrawal threshold in celecoxib and vehicle group. (XLSX)

S3 Table Withdrawal threshold in NSAIDs, acetaminophen and vehicle group. (XLSX)

S4 Table Area under the curve in NSAIDs, acetaminophen and vehicle group. (XLSX)
